# Chemical and Cellular Assays Combined with *In Vitro* Digestion to Determine the Antioxidant Activity of Flavonoids from Chinese Bayberry (*Myrica rubra* Sieb. et Zucc.) Leaves

**DOI:** 10.1371/journal.pone.0167484

**Published:** 2016-12-02

**Authors:** Yu Zhang, Shiguo Chen, Chaoyang Wei, Hui Gong, Lanqi Li, Xingqian Ye

**Affiliations:** Zhejiang University, Department of Food Science and Nutrition, Zhejiang Key Laboratory for Agro-Food Processing, Fuli Institute of Food Science, Zhejiang R & D Center for Food Technology and Equipment, Hangzhou, China; Islamic Azad University Mashhad Branch, ISLAMIC REPUBLIC OF IRAN

## Abstract

Oxidative stress is highly associated with the development of cardiovascular diseases and cancer and has drawn great attention. Natural products suggest a potential role in prevention of these disorders. The aim of this study was to investigate the antioxidant and anti-cancer properties of Chinese bayberry leaves (*Myrica rubra* Sieb. et Zucc.) flavonoids (BLF) comprehensively through the combination of *in vitro* digestion, chemical and cellular antioxidant assays. Based on the LC/MS data, the major flavonoids of BLF were myricitrin and quercetin 3-rhamnoside. BLF owned strong chemical and cellular antioxidant activity (CAA) with its CAA value at 4253.884 ± 435.366 μmol of QE/100 g DW. After the *in vitro* digestion, the total flavonoids content, myricitrin and quercetin 3-rhamnoside decreased significantly (P < 0.05). Lower levels of the total flavonoid content and cellular uptake of myricitrin and quercetin 3-rhamnoside might contribute to the lower CAA value of digested BLF (DBLF). However, DBLF still owns considerable chemical antioxidant activities and CAA compared with many plants. Furthermore, both BLF and DBLF exhibited dose-dependent relationship against HepG2 proliferation. Taken together, BLF has a great potential to be developed as a natural antioxidant for promoting public health.

## Introduction

Cardiovascular diseases and cancer are the leading causes of death all over the world [1] and oxidative stress is highly associated with their development. Oxidative stress refers to the imbalance between the products of reactive oxygen species (ROS) and the biological ability to detoxify them [2]. The causes of oxidative stress might be (a) the increasing level of ROS due to the products from toxins metabolized to ROS; (b) the excessive activation of ROS systems; (c) the deficient antioxidant defense [3]. Generated ROS, such as superoxide radical (O_2_^-^), hydroxyl radical (OH) and hydrogen peroxide (H_2_O_2_) might induce damage to base, DNA strands, as well as other components in cells, such as proteins and lipids [4]. Extensive studies [5] have demonstrated the ability of phytochemicals from fruit and vegetables to scavenge free radicals and the importance of phytochemicals in promotion of antioxidant and anti-cancer ability in the body. Therefore, consumption of phytochemicals from plants is encouraged to help combat oxidative stress and cancer.

Chinese bayberry (*Myrica rubra* Sieb. et Zucc.) has been cultivated in Southern China for more than 2,000 years [6], and China is also a major country for bayberry output, with the annual amount around 300,000 tons [7]. Leaves from Chinese bayberry trees are luxuriant and remain green throughout a year. However, they are always abandoned after harvest, which causes considerable ecological waste. Some studies [8–10] showed that Chinese bayberry leaves extracts exhibited antioxidant, antimicrobial and antiviral properties. However, these studies failed to specify what bioactive components exerted those functions. Also, studies on the antioxidant and anti-cancer effects of Chinese bayberry leaves towards cancer cells are scarce. Apart from chemical antioxidant assays, such as DPPH, ABTS, FRAP and ORAC, the cellular antioxidant assay developed by Wolfe and Liu [3] could assess the antioxidant capacity of phytochemicals under the complex biological system because it takes cellular distribution and bioavailability in account and therefore it can better predict the antioxidant capacity of phytochemicals.

It is well known that polyphenols including flavonoids and phenolic acids are subjected to structural modification after ingestion and gastrointestinal digestion due to the acid and alkaline conditions and their bioactivities might be largely changed due to such modification [5]. Therefore, in order to evaluate the antioxidant ability of flavonoids from Chinese bayberry leaves comprehensively, *in vitro* digestion combined with chemical antioxidant assays (DPPH, FRAP and ORAC) and cellular assay (cellular antioxidant assay and cellular uptake of flavonoids) were used in the present study. The antiproliferative property of BLF was further investigated as well. Changes of total flavonoids content (TFC) and major components were compared before and after the *in vitro* digestion. Particularly, cellular uptake of major bioactive components was quantified before and after the *in vitro* digestion in order to elucidate clearer antioxidant mechanism of flavonoids from Chinese bayberry leaves.

## Materials and Methods

### 2.1 Ethic statement

We contact a local farmer who owned a field of bayberry trees in Cixi and harvested the leaves under his permission. In this case, no conflicts of interests are existed and no endangered or protected species are included. The specific location is around 30.075315, 121.562494.

### 2.2 Chemicals and reagents

Leaves of Chinese bayberry Biqi were picked in Cixi, Zhejiang, China in September 2015. HPD-500 was purchased from Solarbio Life Sciences (China). Methanol and formic acid for HPLC analysis were of HPLC grade (Purity ≥ 99.9%). All other reagents and solvents used were of analytical grade, unless stated otherwise. 6-hydroxy-2,5,7,8-tetramethyl-chroman-2-carboxylicacid (Trolox), 2,2’-Azobis (2- methyl propionamidine) dihydrochloride (AAPH), 2,2-diphenyl-1-picrylhydrazyl (DPPH), 2,2’-azino-bis-(3-ethylbenzo-thiazoline-6-sulphonic acid) diammonium salt (ABTS), 3-(4,5-dimethylthiazol-2-yl)-2,5-diphenyltetrazolium bromide (MTT), 2,4,6-tri(2-pyridyl)-1,3,5-triazine (TPTZ), 2’,7’-dichlorofluorescin diacetate (DCFH -DA), fluorescein disodium salt, α-amylase, pepsin lipase, bile salts, Folin–Ciocalteu reagent, gallic acid, rutin, myricitrin and quercetin 3-rhamnoside were purchased from Sigma–Aldrich (St. Louis, MO, USA). Dulbecco’s modified Eagle’s medium (DMEM) and foetal bovine serum (FBS) were purchased from HyClone (Logan, UT, USA).

### 2.3 Sample preparation

Chinese bayberry leaves were dried for 12 h at 40°C and afterwards, 1 kg of the finely ground powder was extracted with 10 L of 70% acetone and washed with hexane and dichloromethane for 12 h at room temperature. The extraction was performed three times. Organic solvent was removed by rotary evaporation and the aqueous phase was lyophilized to dryness to yield crude powder, which was then purified through a HPD-500 column and eluted with ethanol to remove sugar. Afterwards, the purified powder was further purified by a Sephadex LH-20 (300 mm × 30 mm i.d.) column and eluted with 90% methanol. After rotary evaporation under vacuum and lyophilization to dryness, Chinese bayberry leaves flavonoids powder was obtained labeled as BLF.

### 2.4 *In vitro* gastrointestinal digestion

The *in vitro* digestion method was performed based on previous methods with slight modification [11, 12]. Briefly, 2 g of BLF powder was added to 10 mL of distilled water and mixed with 100 μL of α-amylase (6.5 mg of α-amylase dissolved in 5 mL of 1 mM CaCl_2_ at pH 7.0) and the mixture was incubated in a water bath at 37°C for 10 min. Then, the mixture underwent the gastric digestion step and the pH value of the mixture was adjusted to 2.0 with 6 N HCl. Pepsin was added to the mixture at the concentration of 0.1 g of pepsin/g of sample and was incubated in a water bath at 37°C for 1 h. Then the pH was adjusted to 6.0 with 0.9 M NaHCO_3_. Afterwards, 5 mL of lipase-bile (0.1 g lipase and 0.625 g of bile salts dissolved in 25 ml of 0.1 M NaHCO_3_) was added to the mixture. The pH of the digesta was readjusted to 7.5 with 0.9 M NaHCO_3_, and the mixture was incubated at 37°C for 2 h. The disgesta was then centrifuged at 10,000 rpm for 10 min and the supernatants were obtained and diluted to 100 mL. The diluted solution was then kept at -80°C until analysis and was labeled as digested bayberry leaves flavonoids (DBLF).

### 2.5 Determination of phenolic compounds

Determination of total flavonoids content (TFC) was based on a previous method with slight modification [13]. Briefly, 100 μL of diluted BLF at 0.2 mg/mL and DBLF at 0.5 mg/mL were mixed with 300 μL of 5% aqueous NaNO_2_ completely and stood for 5 min. Then 300 μL of 10% aqueous Al(NO_3_)_3_ was added to the mixture and stood for 6 min. 4 mL of 1 M NaOH was added afterwards and 400 μL of 30% ethanol was added to make the final volume at 10 mL. The mixture solution was fully vortexed and stood for 10 min. 50% aqueous methanol was used as a reagent blank. Finally, the absorbance was measured against the blank at 510 nm. The TFC was calculated with respect to a rutin standard curve and results were represented as milligrams per gram of rutin equivalent.

HPLC-UV-ESIMS analysis was performed on a Waters platform, including a Waters 2695 separation module, a Waters 1525 pump, a Waters 2487 detector and was coupled with a Bruker Esquire 3000 Plus ion trap mass spectrometer (Bruker-Franzen Analytik GmbH, Bremen, Germany) equipped with ESI. Mass Spectra were achieved by electrospray ionization in the negative mode. Separation was carried out on a Zorbax SB-C18 (Agilent, USA) column (250 × 4.6 mm, 5 μm). The mobile phase consisted of (A) 0.1% formic acid solution and (B) methanol. The elution program was as follow: 0–7 min, 10–20% B; 7–17 min, 35% B; 17–37 min, 35–90% B. The flow-rate was 1.0 ml/ min and the column temperature was 30°C. The injection volume was 10 μL and UV detection wavelengths were set at 365 nm. Cellular uptake of BLF and DBLF was determined based on the similar conditions as mentioned above and the injection volume was 50 μL.

The phenolic compounds were identified mainly by comparing them with published data by their UV-Vis spectra and ESIMS spectra. If relevant standards were available, they were also identified by comparing the chromatography with the authentic standards.

### 2.6 Chemical antioxidant activities

#### 2.6.1 DPPH assay

The DPPH assay was performed based on a previous method by a previous report [14] with slight modification. Briefly, 0.1 mL of diluted BLF or DBLF were added into 3.9 mL of 0.1 mM DPPH in ethanol. Ethanol was used as the reagent blank. The mixture solution was kept at room temperature in the dark for 20 min and the absorbance was measured at 517 nm. Results were expressed as milligrams of Trolox equivalents (TE) per gram DW.

#### 2.6.2 FRAP assay

The FRAP assay was determined based on a previous report [15] with slight modification. The FRAP reagent consisted of 10 mM TPTZ (dissolved in 40 mM HCl), 20 mM ferric chloride and 0.1 mol/L acetate buffer (pH 3.6) at a ratio of 1:1:10 (v/v/v). 100 μL of samples reacted with 3.9 mL of prepared FRAP solution for 10 min at 37°C in the dark, and then the absorbance was measured at 593 nm. The results were expressed as milligrams of Trolox equivalents (TE) per gram DW.

#### 2.6.3 ORAC assay

The ORAC assay was performed according to a previous method [16] with slight modification. The fluorescein solution was prepared with 75 mmol/L potassium phosphate buffer (pH 7.4) to a final concentration of 504 nmol/L. Afterwards, 25 μL of sample solution and 25 μL of fluorescein solution were added to a black bottom 96-well plate, which was kept in a Thermo-Shaker (MB100-2A) at 37°C for 5 min followed by the addition of 150 μL of 17.07 mmol/L AAPH potassium phosphate buffer solution. After thoroughly shaking for 5s, absorption measurements were recorded every 1 min for 3 h at 37°C using a Fluoroskan Ascent FL plate-reader (Thermo Fisher Scientific, USA) with 538 nm as the emission wavelength and 485 nm as the excitation wavelength. Results were expressed as milligrams of TE per gram DW.

### 2.7 Cell culture

HepG2 cells were purchased from American Type Culture Collection (ATCC, VA, USA) and were incubated in DMEM solution with 10% FBS, 50 μg/mL of streptomycin and 50 units/mL of penicillin. The cells were kept in an incubator with 5% CO_2_ at 37°C. The cells used in this study were between passages 12 and 35.

### 2.8 Cytotoxicity and cellular antioxidant activity (CAA) assay

Cytotoxicity was performed based on a previous method [12]. HepG2 cells were seeded on 96-well flat-bottom plates at a concentration of 4 × 10^4^ cells/well and incubated for 24 h at 37°C. The medium was removed and the cells were washed with phosphate buffer (PBS). Cells were incubated with 100 μL of BL/DBLF at different concentrations and 100 μL of treatment media (DMEM without FBS) for another 24 h at 37°C and 5% CO_2_. Afterwards, detection agent (MTT) was added to each well and the cells were incubated for 4 h. The dye was removed and 100 μL of dimethyl sulfoxide was added to dissolve cells. After shaking for 10 min, the plate was measured at 550 nm in a 96-well plate reader (iMark, Bio- Rad, USA). The sample concentrations whose absorbance readings decreased by less than 10% compared to the control were considered not cytotoxic and used in the CAA assay.

The CAA assay was performed based on the previous method [3]. In brief, HepG2 cells were seeded at black 96-well microplates (with transparent bottoms) in 100 μL growth media at a density of 6 × 10^4^ cells/well and incubated for 24 h at 37°C. The media was then removed and the cells were washed with PBS. 100 μL of medium containing BLF and DBLF at different concentrations and 25 μM of DCFH-DA for 1 h were added to the cells and incubated for 1 h. Then, 100 μL of 600 μM AAPH solution (dissolved in HBSS) was added to the cells. The plate was placed to a Fluoroskan Ascent FL plate-reader (Thermo Fisher Scientific, USA) and measured at 538 nm (emission wavelength) and 485 nm (excitation wavelength) every 2.5 min for 1 h. After blank subtraction from the fluorescence readings, the area under the curve of fluorescence versus time was integrated to calculate the CAA value at different concentrations of samples as follows:

CAA=(1−∫SA/∫CA)×100

where ∫SA is the integrated area under the sample fluorescence versus time curve, while ∫CA is the integrated area obtained from the control group curve. The median effective dose (EC50) was determined for samples from the median effect plot of log (fa/fu) versus log (dose), where fa is the fraction influenced (CAA unit) and fu is the fraction uninfluenced (1-CAA unit) by the treatment. Quercetin was used as the standard and the results were expressed as mmol of quercetin equivalents (QE) per 100 grams (DW). The EC50 values are given as the mean ± SD of data from three separate experiments.

### 2.9 Cellular uptake of flavonoids of Chinese bayberry leaves towards HepG2 cells

To further clarify which component played a major role in CAA, cellular uptake of BLF and DBLF was performed based on a previously described method with slight modification [17]. HepG2 cells were incubated with 4 mL of samples at 0.5 mg/mL (no cytotoxicity) and 4 mL of DMEM for 30 min with 5% CO_2_ at 37°C. The treatment was removed and the cells were washed for three times. Afterwards, cells were scraped in acidified methanol (pH 2.0) and the wells were rinsed three times with methanol. Ultrasound treatment was then used to break the cells for 20 min. The mixture was centrifuged at 10,000 rpm for 5 min and the supernatant was collected. Then cells were washed three times more to ensure complete extraction and all of the supernatant was collected and were evaporated to dryness under nitrogen. The residue was redissolved in 500 μL of methanol for HPLC analysis

### 2.10 Inhibition of HepG2 cell proliferation

The antiproliferative activities of BLF and DBLF against HepG2 cells were assessed by a previous method [18]. Briefly, cells were seeded at the density of 2.5 × 10^4^ cells/well on 96-well plates. After a 4 h incubation with 5% CO_2_ at 37°C, cells were washed with PBS and 100 μL of samples at different concentrations and 100 μL of growth media were added. Control cultures received the extraction solution without the samples and blank wells contained 100 μL of growth medium with no cells. After a 72-h incubation, cell proliferation was determined by the methylene blue assay [19]. The percentage of cell proliferation of samples was determined from the absorbance reading at 570 nm compared to the control. The antiproliferative activity was expressed as EC_50_ values. At least three replicates were done for each sample to determine the antiproliferative activity.

### 2.11 Statistical analysis

Data from the present study were presented as mean ± SD for at least three replicates for each sample. Microsoft Excel 2007 and SPSS Program, version 9.5.0.0 (IBM SPSS Statistics 23) were used for regression analyses and other statistical analyses. Means were compared by Duncan’s new multiple range test. Statistically significant differences were set at p < 0.05.

## Results and Discussion

### 3.1 Phenolic profile of BLF and DBLF

**Fig 1:**
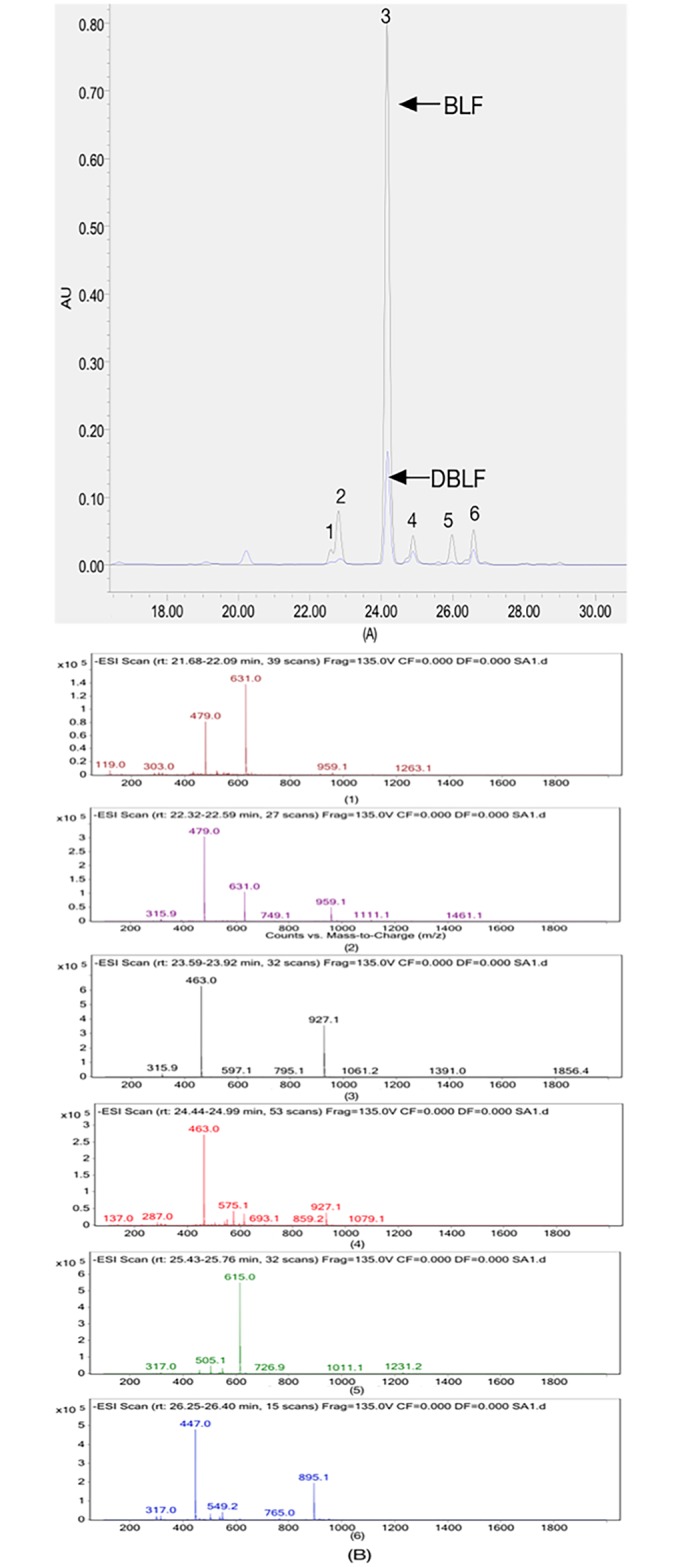
HPLC chromatograph of BLF and DBLF and ESIMS spectra of BLF. (A) HPLC chromatograph detected at 365 nm of BLF and DBLF. (B) ESIMS spectra (1): peak 1; (2): peak 2; (3): peak 3; (4): peak 4, (5): peak 5 and (6): peak 6 of BLF.

Flavonoids in Chinese bayberry leaves were separated via the HPLC separation elution and five peaks were shown in Table 1 and Fig 1. Kim *et al*. [20] and Yang *et al*. [21] also reported flavonoids from bayberry leaves. Based on the data of their studies, flavonoids from the present study could be tentatively identified and authentic standards were also used to assure the identification. Peak 1 owned its molecular ion [M-H]^-^ at *m/z* 631 and duple molecular ion [2M-H]^-^ at *m/z* 1263.1, which were similar as the data of myricetin 3-O-(2”-O-galloyl)-β-D-galactopyranoside [20]. Peak 2 had its molecular ion [M-H]^-^ at *m/z* 479 and duple molecular ion [2M-H]^-^ at *m/z* 959.1 and it also showed a fragment ion at *m/z* 315.9, which was a typical mass in the negative mode of myricetin aglycone, indicating that it was a myricetin derivatives [21]. Therefore, it was tentatively identified as myricetin hexoside. Peak 3 had its molecular ion [M-H]^-^ at *m/z* 463 and duple molecular ion [2M-H]^-^ at *m/z* 927.1, which were in accordance with the information of myricitrin. Also, its retention time was perfectly matched with the myricitrin standard and therefore it was identified as myricitrin. Based on the molecular ion and duple molecular ion data, peak 4 and peak 5 were tentatively identified as myricetin deoxyhexoside and myricetin deoxyhexoside-gallate, respectively [21]. Peak 6 had a molecular ion [M-H]^-^ at *m/z* 447 and its duple molecular ion [2M-H]^-^ at *m/z* 895.1 and its retention time was matched with the quercetin 3-rhamnoside standard. Therefore, the compound was positively identified as quercetin 3-rhamnoside. Since myricitrin and quercetin 3-rhamnoside contributed to the major components of BLF, therefore, their quantification before and after *in vitro* gastrointestinal digestion and of cellular uptake towards HepG2 cells were further investigated.

**Table 1:** Identification of individual flavonoids from Chinese bayberry leaves by using HPLC-UV-ESIMS and respective standards.

Peak No.	RT^b^ (min)	Molecular weight	HPLC-ESIMS (*m/z*)	Tentative identification
1	22.52	632	631, 1263.1	Myricetin-3-O-(2”-O-galloyl)-β-D-galactopyranoside
2	22.54	480	479, 959.1	Myricetin hexoside
3	24.25	464	463, 927.1	Myricitrin (std)^a^
4	24.92	464	463, 927.1,	Myricetin deoxyhexoside
5	26.01	616	615, 1231.2,	Myricetin deoxyhexoside-gallate
6	26.52	448	447, 895.1	Quercetin 3-rhamnoside (std)^a^

^a^The compound was also identified by comparing the chromatography with the authentic standards.

^b^RT refers to HPLC retention time.

The content of myricitrin and quercetin 3-rhamnoside and TFC of BLF before and after *in vitro* digestion were presented in Table 2. The TFC of BLF decreased 27.32% approximately after the *in vitro* digestion. Particularly, the content of myricitrin and quercetin 3-rhamnoside in BLF were 184.36 ± 5.96 mg/g DW and 127.05 ± 0.42 μg/g DW, respectively and they decreased around 80.32% and 56.09% in DBLF. Also, peak 1, peak 2 and peak 5 almost disappeared after the *in vitro* digestion. Such results suggested that BLF was not stable under digestion condition, which was also in accordance with findings in other studies [12, 22]. This might be associated with (a) the interaction between flavonoids and other components, such as digestive enzymes [23]; (b) the conversion of phenolics into other unknown or undetected compounds under dramatic pH changes. Particularly, flavonoids from Chinese bayberry leaves are mainly in the form of glycosides. After experiencing the *in vitro* digestion, they are easily degraded under a mildly alkaline condition to form aglycone [24] or other components [25]. Therefore, both the content of myricitrin and quercetin 3-rhamnoside significantly decreased after the *in vitro* digestion.

**Table 2:** Total flavonoids content (TFC), myricitrin and quercetin 3-rhamnoside content of BLF and DBLF.

	BLF	DBLF
TFC^a^	920.78±18.88^d^	669.18±40.95^d^
Myricitrin^b^	184.36±5.96^d^	36.29±0.35^d^
Quercetin 3-rhamnoside^c^	127.05±0.42^d^	55.79±0.08^d^

^a^Total flavonoids were expressed as milligrams of rutin equivalent per gram DW (P < 0.05).

^b^Myricitrin content was expressed as milligrams of myricitrin equivalent per gram DW (P < 0.05).

^c^Quercetin 3-rhamnoside content was expressed as micrograms of quercetin 3-rhamnoside equivalent per gram DW (P < 0.05).

^d^Data are expressed as mean values ± SD (n = 3).

### 3.2 Antioxidant activities of BLF and DBLF

**Fig 2:**
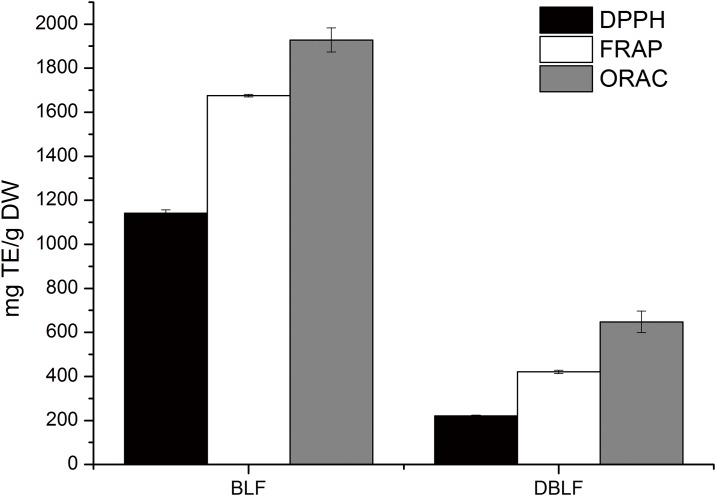
Antioxidant activities determined by DPPH, FRAP and ORAC assays of BLF and DBLF. Their values were expressed as milligrams of TE per gram of DW. Different letters refer to statistically significant differences at p < 0.05, Duncan’s multiple range test.

The chemical antioxidant capacities of BLF and DBLF determined by the DPPH, FRAP and ORAC assays were shown in Fig 2. BLF owned obviously higher antioxidant abilities than those of DBLF (P < 0.05). The DPPH, FRAP and ORAC values of BLF were 859.63 ± 12.85, 1641.07 ± 22.03 and 1927.94 ± 54.90, respectively, and they decreased about 74.32%, 74.37% and 64.59%, respectively (P < 0.05) after the *in vitro* digestion. Similar results were also reported in other studies that *in vitro* digestion contributed to the loss of antioxidant capacity of phenolic compounds [12, 22]. It should be noted that after the *in vitro* digestion, TFC and the content of myricitrin and quercetin-3-rhamnoside decreased significantly (P < 0.05) in BLF. Since phenolic compounds are highly sensitive to the alkaline condition in the small intestine and their structures may undergo modifications, which caused a decreased amount of bioaccessible total polyphenols, flavonoids and anthocyanins [22]. Possible mechanism for BLF to exhibit its antioxidant ability associates with the structure of its major components (myricitrin and quercetin-3-rhamnoside) [26, 27]: (a) the dihydroxyl groups in the B ring own higher stability of aroxyl radicals and are responsible for electron dislocation; (b) the 2,3-double bond in conjugation with a 4-oxo function is responsible for electron delocalization from the B ring; (c) 3- and 5-OH groups as well as the 4-oxo group own potent radical scavenging ability; (d) the presence and the number of hydroxyl groups linked to phenolic rings are related with the increasing radical scavenging ability [28]; (e) the addition of sugar moiety might improve the antioxidant property of the aglycone group (myricetin) [29]. Thus, the specific structures and functional groups attribute to the total antioxidant property of BLF. Although the *in vitro* digestion significantly affected the phenolic profile of BLF, the ORAC value of DBLF was still significantly higher than that of other plants, such as Chinese bayberry (16.05 mg TE/g DW), yerba mate extract (176.57 mg TE/g DW) and Chinese hawthorn (138.95 mg TE/g DW) [12,22,30]. In comparison with other types of plants, BLF owned greater antioxidant potential even if it undergoes *in vitro* digestion.

### 3.3 CAA and cellular uptake of BLF and DBLF

Compared with chemical antioxidant assays, the CAA assay takes the cellular uptake, distribution, bio-accessibility and metabolism of bioactive components into account and therefore provides a better predictor for the antioxidant ability of bioactive compounds [3] and the CAA value and EC50 of BLF and DBLF were presented in Table 3. The EC50 (mg DW/mL) of BLF was more than 10 times lower than that of DBLF suggesting that a much lower amount of BLF was in need in order to provide CAA towards HepG2 cells compared with DBLF. In addition, the CAA value of BLF was more than 7-fold of that of DBLF. *In vitro* digestion imposed different effects on the CAA of phenolic compounds. Huang *et al*. [12] reported that after the *in vitro* digestion, the CAA of four different types of Chinese bayberry decreased obviously (P < 0.05), which was also in accordance with our results. However, Faller *et al*. [31] revealed that after the *in vitro* digestion, CAA of the feijoada (a type of plant food rich meal) increased almost two fold (P < 0.05). These results suggested that the content of components changes and structural modification of phenolic compounds after the *in vitro* digestion played an important role in CAA.

**Table 3:** Antioxidant activity determined by CAA assay and cellular uptake of myricitrin and quercetin 3-rhamnoside of BLF and DBLF.

	BLF	DBLF
EC50 (mg DW/mL)^a^	0.056±0.005^d^	0.417±0.011^d^
CAA value (μmol of QE/100 g DW)	4253.884±435.366^d^	566.786±15.068^d^
Cellular uptake of myricitrin (mg/100 g DW*h)^b^	47.20±5.55^d^	16.75±2.05^d^
Cellular uptake of quercetin 3-rhamnoside (mg/100 g DW*h)^c^	39.45±5.25^d^	15.25±2.90^d^
Cytotoxicity (%)	<10	<10

^a^EC50 values were used to convert to CAA values, expressed as micromoles of QE per 100 gram of DW.

^b^Cellular uptake of myricitrin was expressed as milligrams of myricitrin per 100 gram DW per hour.

^c^Cellular uptake of quercetin 3-rhamnoside was expressed as milligrams of quercetin 3-rhamnoside per 100 gram DW per hour.

^d^Data are expressed as mean values ± SD (n = 3).

To further clarify the major components contributed to the CAA, cellular uptake of components was investigated by HPLC. The cellular uptake of myricitrin and quercetin 3-rhamnoside were 47.20±5.55 and 39.45±5.25 mg/100 g DW*h for BLF and 16.75±2.05 and 15.25±2.90 mg/100 g DW*h for DBLF. The correlation between CAA and the cellular uptake of myricitrin and quercetin 3-rhamnoside were high (R^2^ = 0.995 and 0.986) (P < 0.01), indicating that high content of both myricitrin and quercetin 3-rhamnoside played a vital role in the CAA of BLF. Myricitrin was reported to remain stable under acidic condition corresponding to the gastric environment, but it was degraded easily under alkaline condition corresponding to the intestinal environment. However, study by Yokomizo and Moriwaki [32] showed that digested myricitrin also showed strong inhibition effects against tocopherol and cholesterol oxidation. In the present study, the CAA value of DBLF was higher than that of other fruits or vegetables, such as blueberry, pomegranate and blackberry [5], which suggested the great potential of antioxidant ability of BLF.

### 3.4 Antiproliferation against HepG2 cells of BLF and DBLF

**Fig 3:**
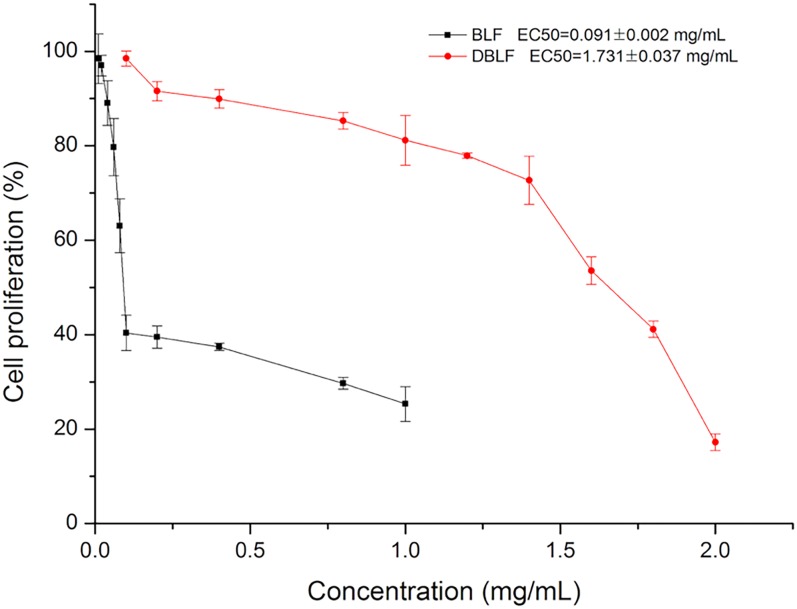
Antiproliferative activity towards HepG2 cells by BLF and DBLF. The curves shown are each from a single experiment (mean ± SD, n = 3).

The antiproliferative ability of BLF and DBLF against HepG2 cells was further investigated in the present study. Both BLF and BDLF could inhibit HepG2 cells proliferation with a dose-dependent manner (Fig 3). The EC50 of BLF and DBLF were 0.091 ± 0.002 and 1.731 ± 0.037 mg/mL, respectively. The EC50 of DBLF was about 19 times higher than that of BLF. Since a lower EC50 represented higher antiproliferative ability, therefore, the antiproliferative ability of BLF was much stronger than that of DBLF. The much higher EC50 and the significant decline of antiproliferative ability might be associated with the decreasing TFC and particularly the content of myricitrin and quercetin 3-rhamnoside. Flavonoids were reported to exhibit anticancer effects through several mechanisms, such as inhibiting procarcinogen molecules, scavenging of electrophilic molecues [33], regulating cancer cell signaling pathways, promotion of apoptosis and modulating enzyme activities [34–36]. Particularly, Xu *et al*. [37] showed that myricitrin was an effective inhibitors of cell proliferation and induced apoptosis in PC-3 cells since it arrested the cell cycle and resulted in the suppression of PC-3 cell growth. The anticancer property of BLF might be related to the functional groups of myricitrin. Shiomi *et al*. [38] reported that the aglycone structure (myricetin) was the key structure to inhibit mammalian DNA polymerase, topoisomerase and human cancer cell proliferation. Edenharder and Grünhage [29] showed that the 3’,4’,5’-trihydroxy structure of myricitrin exhibited stronger antimutagenic ability than those with 3’,4’-dihydroxy structure in ring B. Also, the number of hydroxyl groups in the B ring and the presence of a resorcinol group in the A ring are of great important to inhibit some enzyme activates, such as myeloperoxidase, which might induce inflammatory tissue damage [39].

## Conclusion

Chinese bayberry is popular in China; however, its leaves are usually discarded, which causes a large ecological loss. Here, we combined the *in vitro* digestion with chemical and cellular assays to comprehensively evaluate the antioxidant and antiproliferative properties of BLF in order to further investigate the function and application of BLF. Although the phytochemical content of BLF obviously decreased after the *in vitro* digestion, DBLF still showed strong chemical antioxidant ability and high CAA value compared with other fruits, such as blueberry, pomegranate and blackberry. The cellular uptake of myricitrin and quercetin 3-rhamnoside was strongly associated with the CAA of BLF and DBLF, which suggested that they were the potent bioactive components in BLF. Also, both BLF and DBLF inhibited the proliferation against HepG2 cells in a dose-dependent manner, which might be attributed to the functional structures of myricitrin, such as the 3’,4’,5’-trihydroxy structure and a resorcinol group in the A ring. In conclusion, our results suggested the strong antioxidant and cancer prevention properties of BLF, which might be developed as the natural antioxidant to promote public health.

## Abbreviations

BLF: Chinese bayberry leaves flavonoids
CAA: cellular antioxidant activity
DBLF: digested Chinese bayberry leaves flavonoids
ROS: reactive oxygen species
TFC: total flavonoids content
